# Contrasting Approaches in Advance Care Planning in Dementia as Perceived by General Practitioners

**DOI:** 10.1093/geroni/igaf122.2219

**Published:** 2025-12-31

**Authors:** Jenny van der Steen, Xu Jingyuan, Willemijn Tros, Jeanet W Blom

**Affiliations:** Leiden University Medical Center, Leiden, Zuid-Holland, Netherlands; Leiden University Medical Center, Leiden, Zuid-Holland, Netherlands; Leiden University Medical Center, Leiden, Zuid-Holland, Netherlands; Leiden University Medical Center, Leiden, Zuid-Holland, Netherlands

## Abstract

Various approaches to advance care planning are in use and generally acceptable also for persons with dementia and their care partners. Two contrasting approaches involve a highly scripted, predominantly medical approach to decide on specific treatments in advance versus a more flexible psychosocial, coping-based approach that comprises global care goal setting. We aim to assess for whom and when either approach is preferred from the perspective of general practitioners. General practitioners in the Netherlands participated in the CONT-END randomized controlled trial and we interviewed six practitioners trained in the medical approach and four in the psychosocial approach. We explained the other approach during the interview. Twelve other general practitioners were interviewed after viewing video vignettes of the two approaches shown in random order. With inductive qualitative content analyses, we identified four attributes distinguishing situations in which either approach is preferred: understanding, trust, readiness and momentum. For the medical approach, understanding, trust, and readiness on the part of person and care partner were prerequisites for the right momentum. This may be achieved in a follow-up conversation, but such conversations may also be triggered by urgent medical reasons. In contrast, the psychosocial approach would help understand the person, foster trust and create readiness also in a first conversation early in the course of the disease. Without a clear trigger, momentum would need to be created. We will visualize the contrasting attributes and discuss implications for practice of advance care planning with persons with dementia and their care partners in primary care settings.

